# Supplementary material for: Sesquiterpenes from Artemisia annua and Their Cytotoxic Activities
Source: Molecules. 2022 Aug 10;27(16):5079. doi: 10.3390/molecules27165079 (PMC9414659; doi:10.3390/molecules27165079)
Supplement: Supplementary file 1 [file molecules-27-05079-s001.zip › molecules-1845823-supplementary.pdf]

Sesquiterpenes from *Artemisia annua* and their cytotoxic activities

Xiao Han [1], Yao Chai [2], Cheng Lv [1], Qianqian Chen [1], Jingling Liu [1], Yongli Wang [1,3,*] and Guixin Chou [1,3,*]

Affiliations

[1]The MOE Key Laboratory of Standardization of Chinese Medicines and SATCM Key Laboratory of New Resources and Quality Evaluation of Chinese Medicines, Institute of Chinese Materia Medica (ICMM), Shanghai University of Traditional Chinese Medicine (SHUTCM), China.

[2] Shanghai University (SHU), Shanghai, China.

[3]Shanghai R&D Center for Standardization of Chinese Medicines, Shanghai 201203, PR China

Correspondence

Prof. Guixin Chou Institute of Chinese Materia Medica

Shanghai University of Traditional Chinese Medicine (SHUTCM)

1200 Cai Lun Road, Pudong New Area, Shanghai, China.

chouguixin@shutcm.edu.cn

Mrs. Yongli Wang

Institute of Chinese Materia Medica

Shanghai University of Traditional Chinese Medicine (SHUTCM)

1200 Cai Lun Road, Pudong New Area, Shanghai, China.

ylwang521@126.com

Contents

Figure S1. The HRESIMS spectrum of compound **1**.

Figure S2. The ^{1}H NMR spectrum of compound **1** (MeOD, 400 MHz).

Figure S3. The ^{13}C NMR spectrum of compound **1** (MeOD, 100 MHz).

Figure S4. The HSQC spectrum of compound **1** (MeOD, 400 MHz).

Figure S5. The HMBC spectrum of compound **1** (MeOD, 400 MHz).

Figure S6. The COSY spectrum of compound **1** (MeOD, 400 MHz).

Figure S7. The NOESY spectrum of compound **1** (MeOD, 400 MHz).

Figure S8. The HRESIMS spectrum of compound **2**

Figure S9. The ^{1}H NMR spectrum of compound **2** (MeOD, 400 MHz).

Figure S10. The ^{13}C NMR spectrum of compound **2** (MeOD, 100 MHz).

Figure S11. The HSQC spectrum of compound **2** (MeOD, 400 MHz).

Figure S12. The HMBC spectrum of compound **2** (MeOD, 400 MHz).

Figure S13. The COSY spectrum of compound **2** (MeOD, 400 MHz).

Figure S14. The NOESY spectrum of compound **2** (MeOD, 400 MHz).

Figure S15. X-ray crystallographic structure of compound **3**

Figure S16. Results for DP4+ analysis of compound **1** with ^{13}C NMR chemical shifts calculated at mPW1PW91/6-31G (d, p) level, (isomer 1 = 6*S**, isomer 2 = 6*R**).

Figure S17. Correlation coefficients and DP4+ analysis between experimental and calculated ^{13}C NMR chemical shifts of isomer 1 = 6*S** and isomer 2 = 6*R**

Figure S18. Results for DP4+ analysis of compound **2** with ^{13}C NMR chemical shifts calculated at mPW1PW91/6-31G (d, p) level, (isomer 1 =6*R**, 7*R**, isomer 2 = 6*R**, 7*S**, isomer 3 = 6*S**, 7*R**, isomer 4 = 6*S**, 7*S**).

Figure S19. Correlation coefficients and DP4+ analysis between experimental and calculated ^{13}C NMR chemical shifts of isomer 1 =6*R**, 7*R**, isomer 2 = 6*R**, 7*S**, isomer 3 = 6*S**, 7*R**, and isomer 4 = 6*S**, 7*S**.

Figure S1. The HRESIMS spectrum of compound **1**.

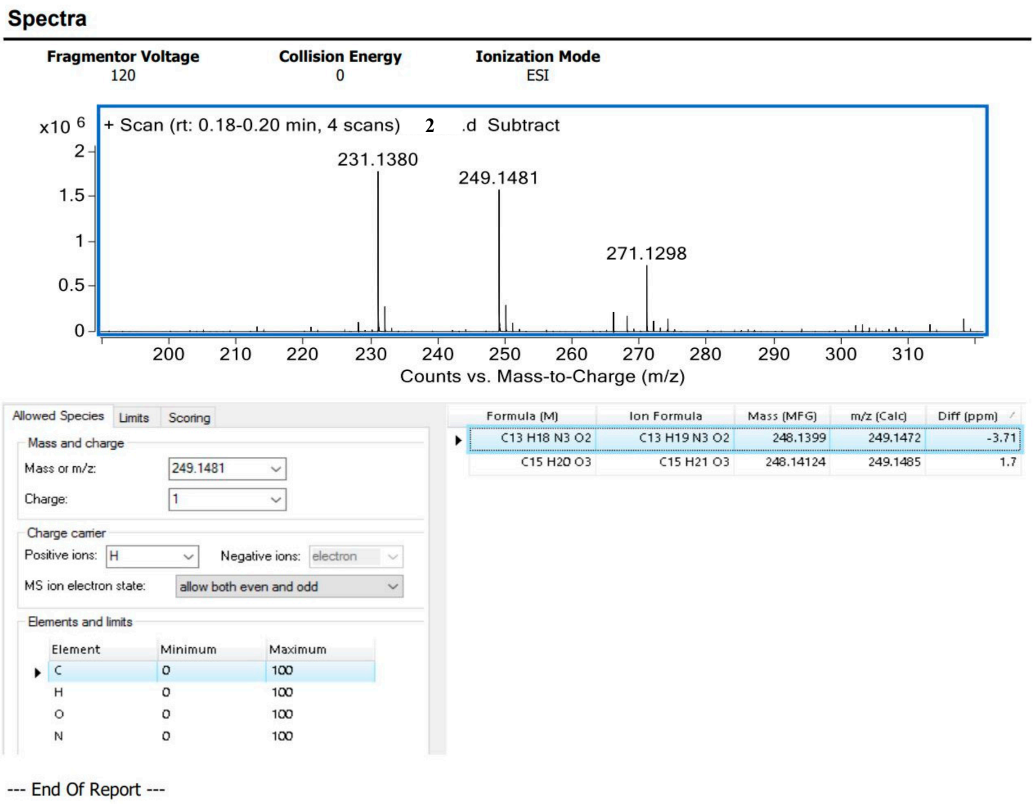

Figure S2. The ^{1}H NMR spectrum of compound **1** (MeOD, 400 MHz).

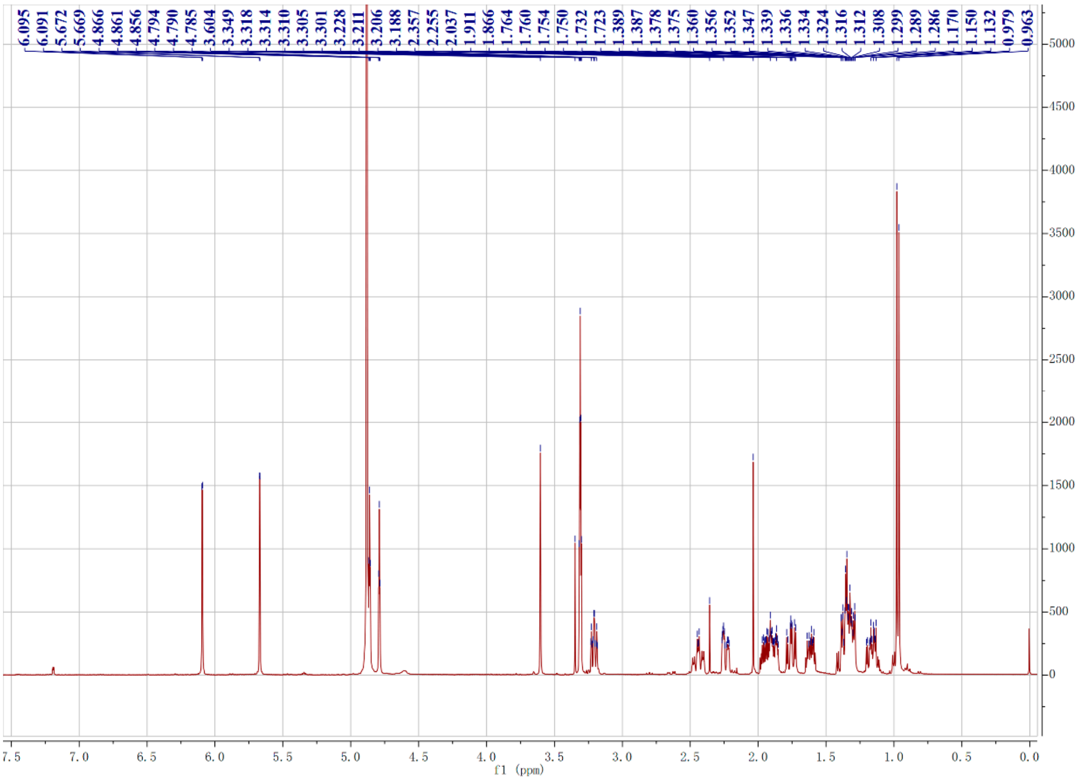

Figure S3. The ^{13}C NMR spectrum of compound **1** (MeOD, 100 MHz).

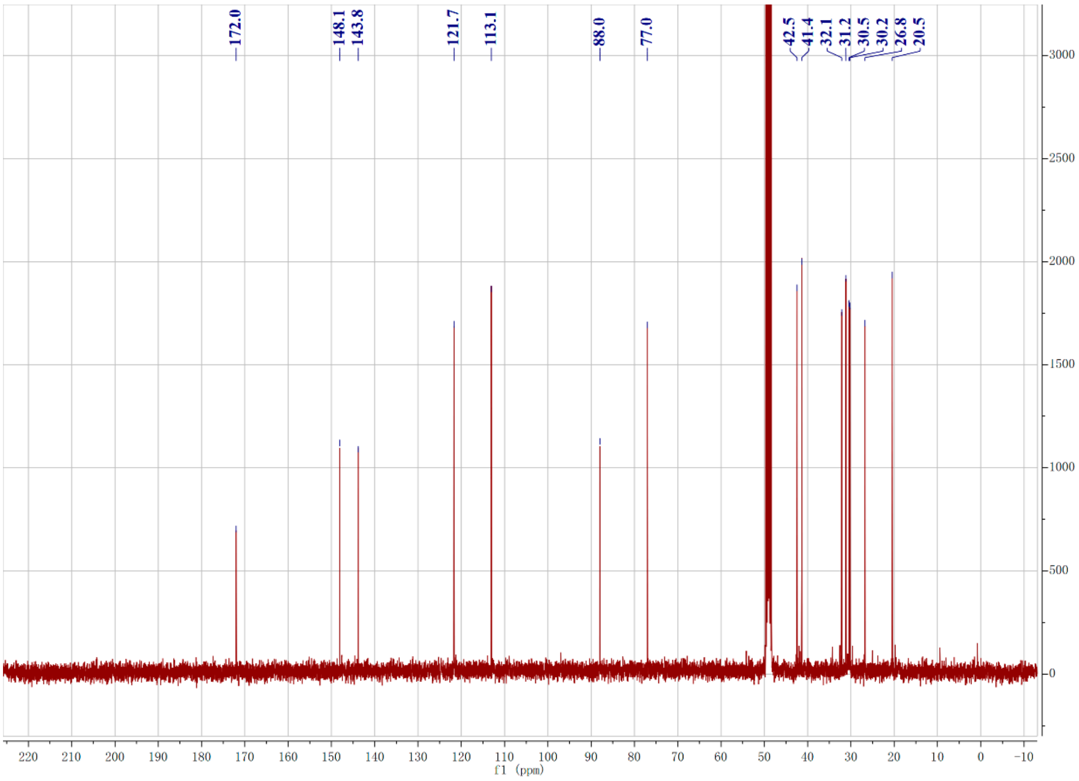

Figure S4. The HSQC spectrum of compound **1** (MeOD, 400 MHz).

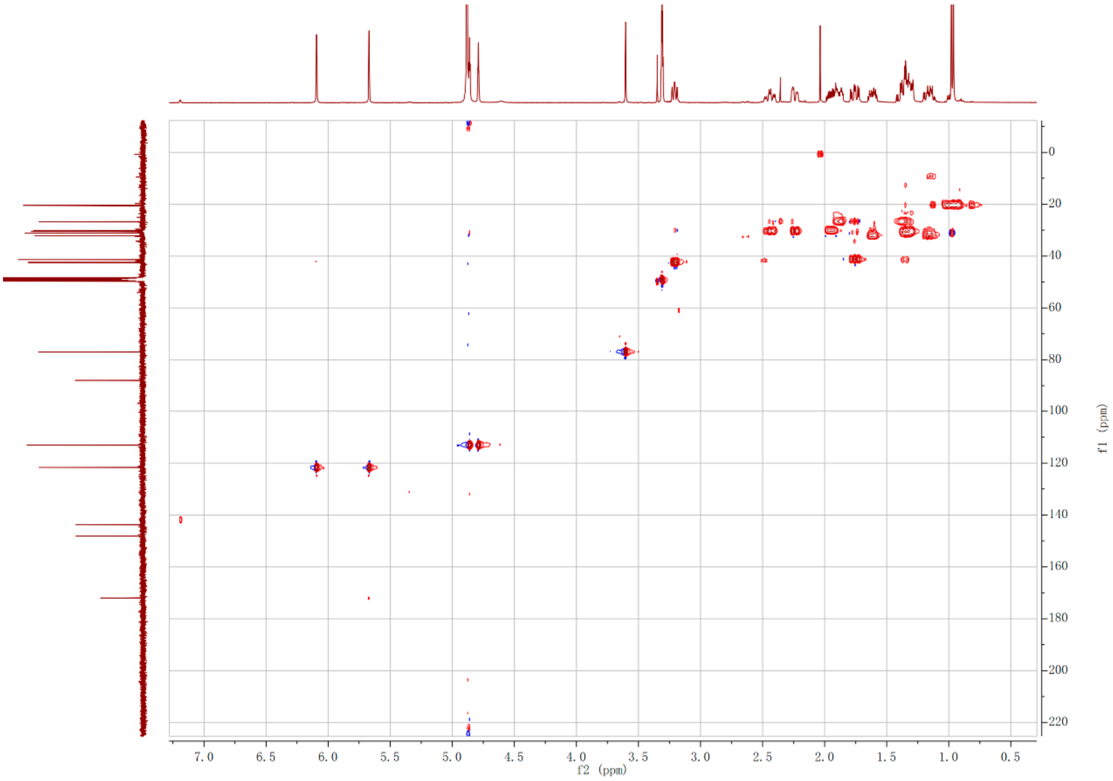

Figure S5. The HMBC spectrum of compound **1** (MeOD, 400 MHz).

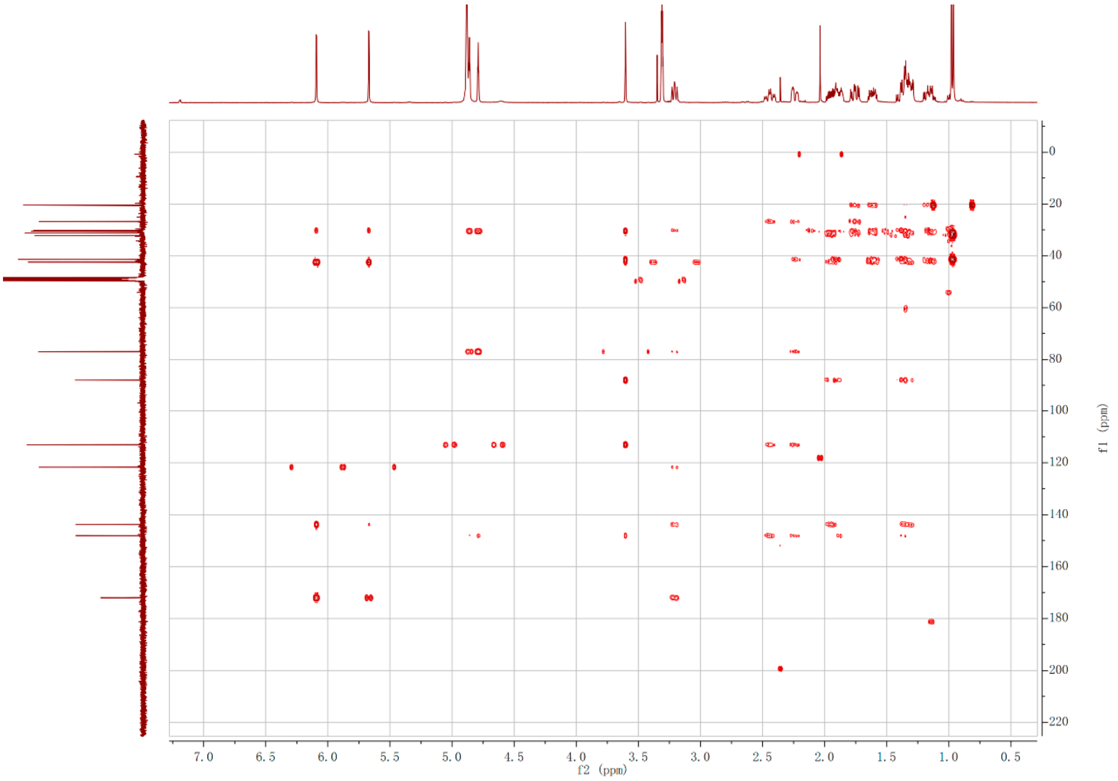

Figure S6. The COSY spectrum of compound **1** (MeOD, 400 MHz).

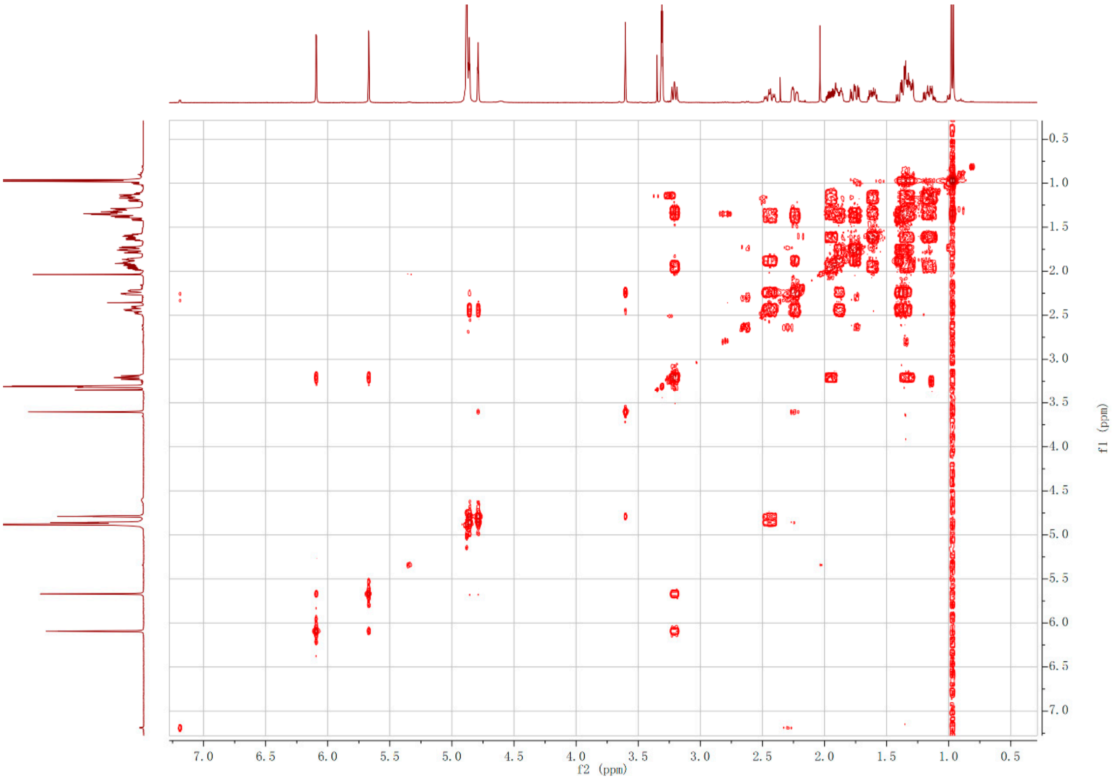

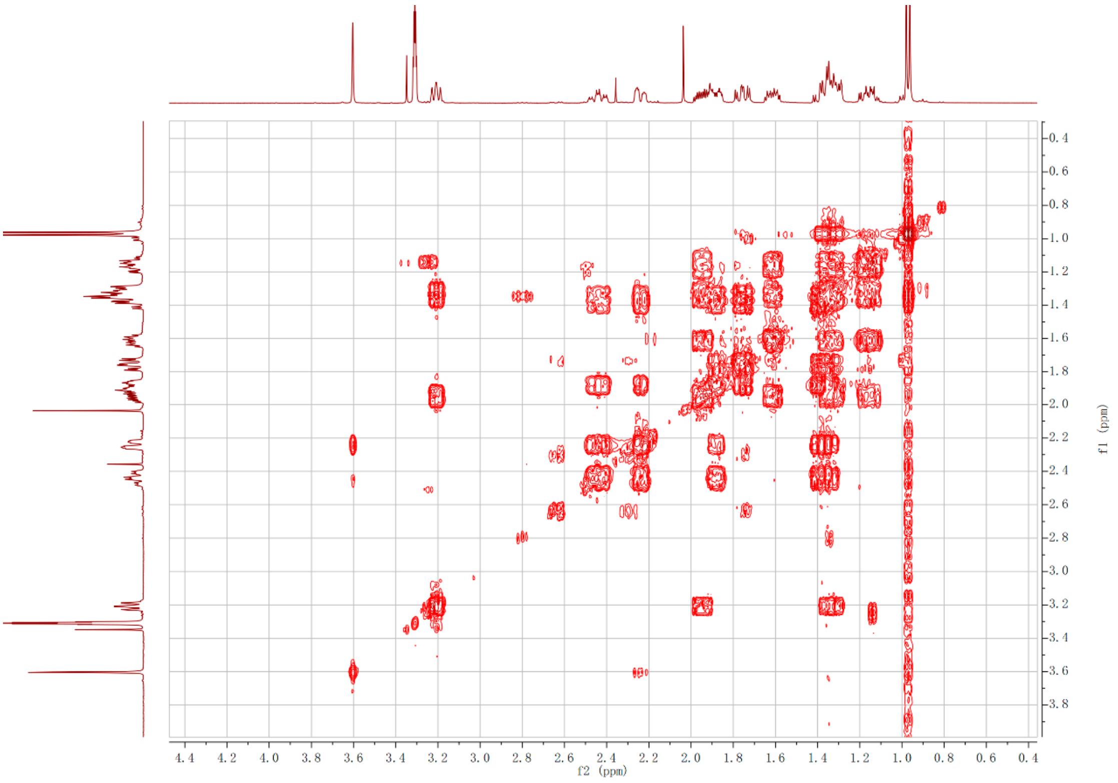

Figure S7. The NOESY spectrum of compound **1** (MeOD, 400 MHz).

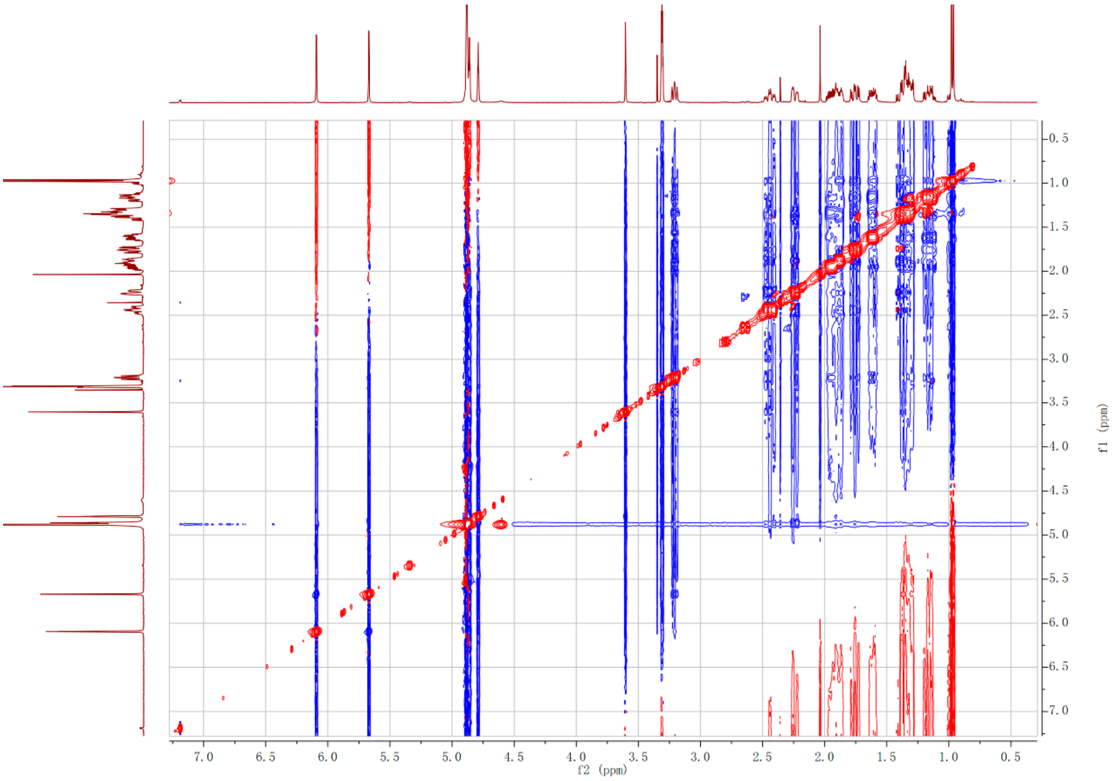

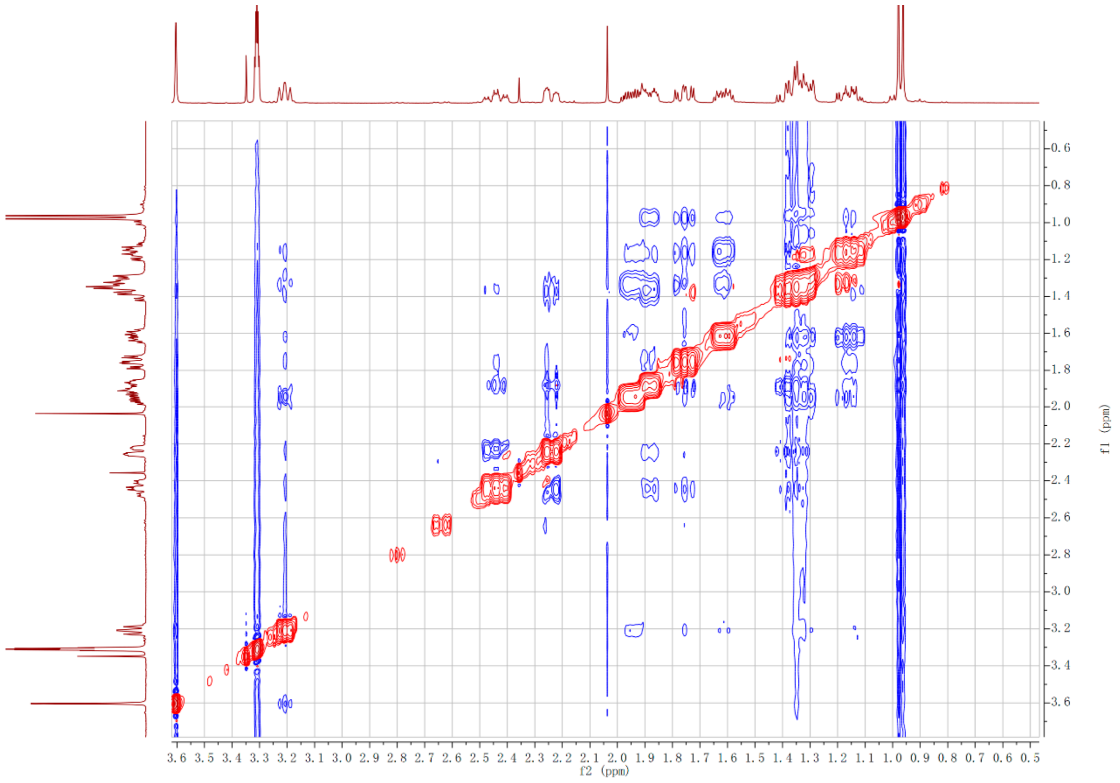

Figure S8. The HRESIMS spectrum of compound **2**

Spectra

Fragmentor Voltage	Collision Energy	Ionization Mode
120	0	ESI

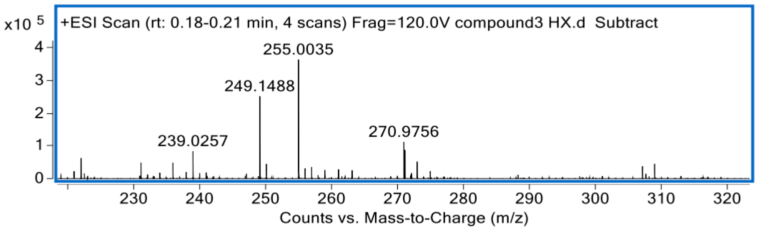

Allowed Species | Limits | Scoring

Mass and charge

Mass or m/z: 249.1488

Charge: 1

Charge carrier

Positive ions: H   Negative ions: H

MS ion electron state: allow both even and odd

Elements and limits

Element	Minimum	Maximum
C	0	100
H	0	100
O	0	100
N	0	100

Formula (M)	Ion Formula	Mass	m/z (Calc)	Diff (ppm)
C15 H20 O3	C15 H21 O3	248.1415	249.1485	-1.12

--- End Of Report ---

Figure S9. The ^{1}H NMR spectrum of compound **2** (MeOD, 400 MHz).

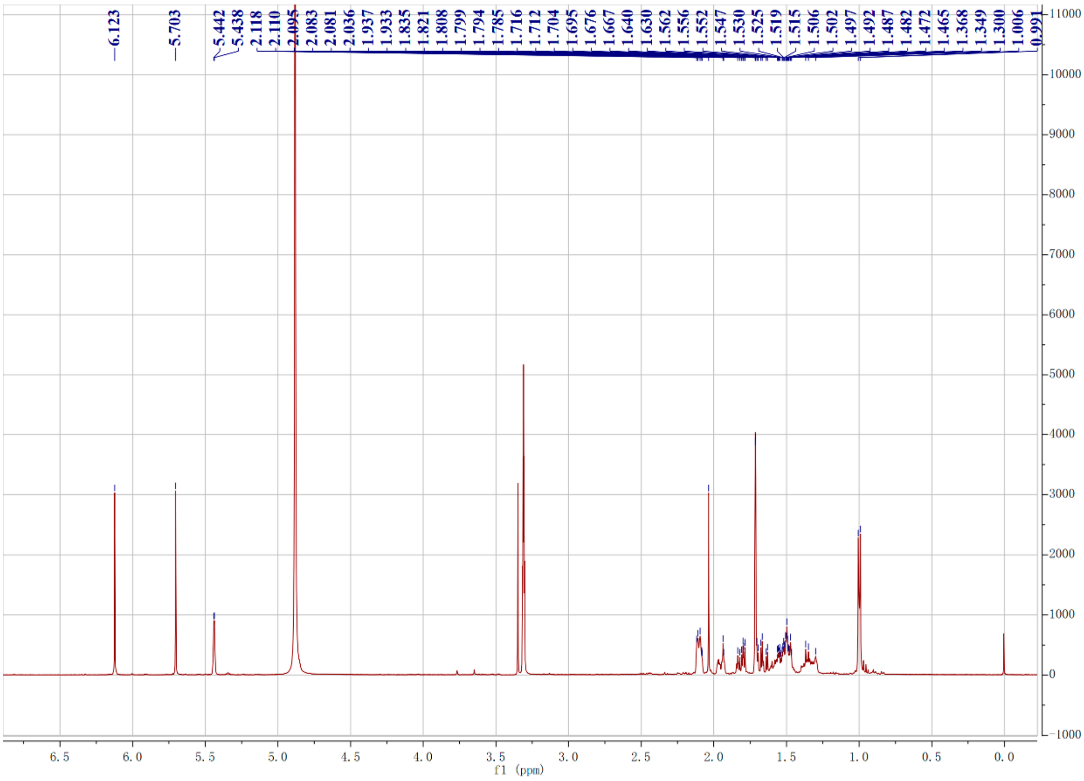

Figure S10. The ^{13}C NMR spectrum of compound **2** (MeOD, 100 MHz).

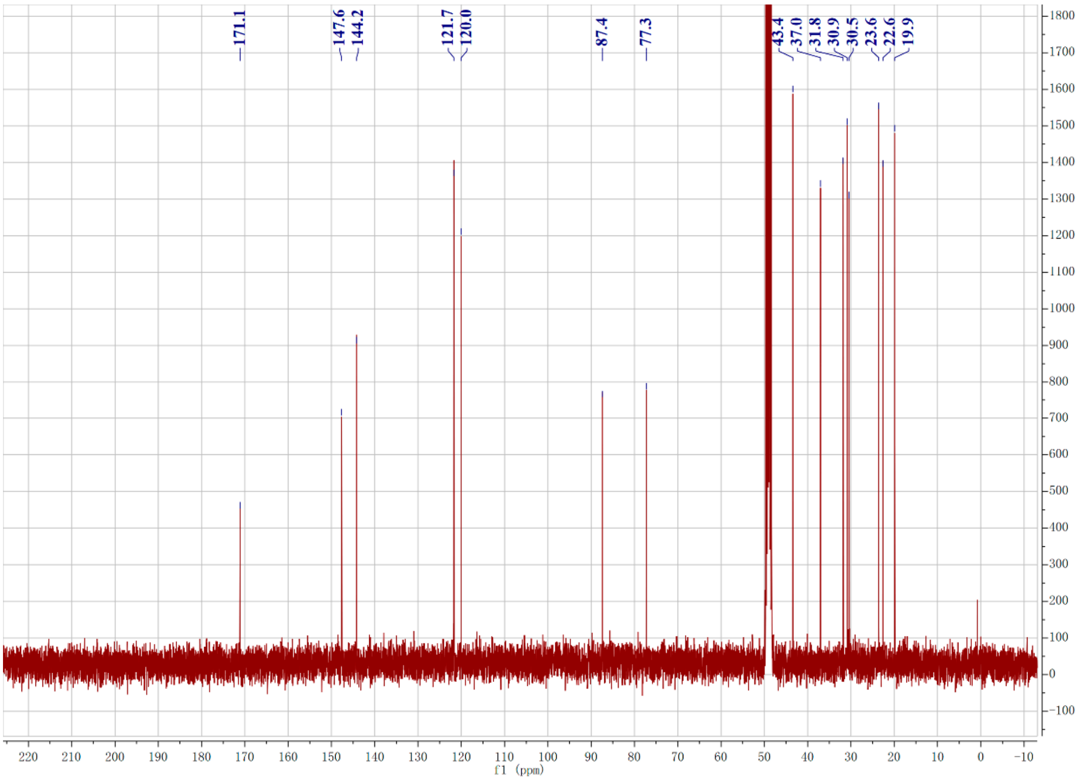

Figure S11. The HSQC spectrum of compound **2** (MeOD, 400 MHz).

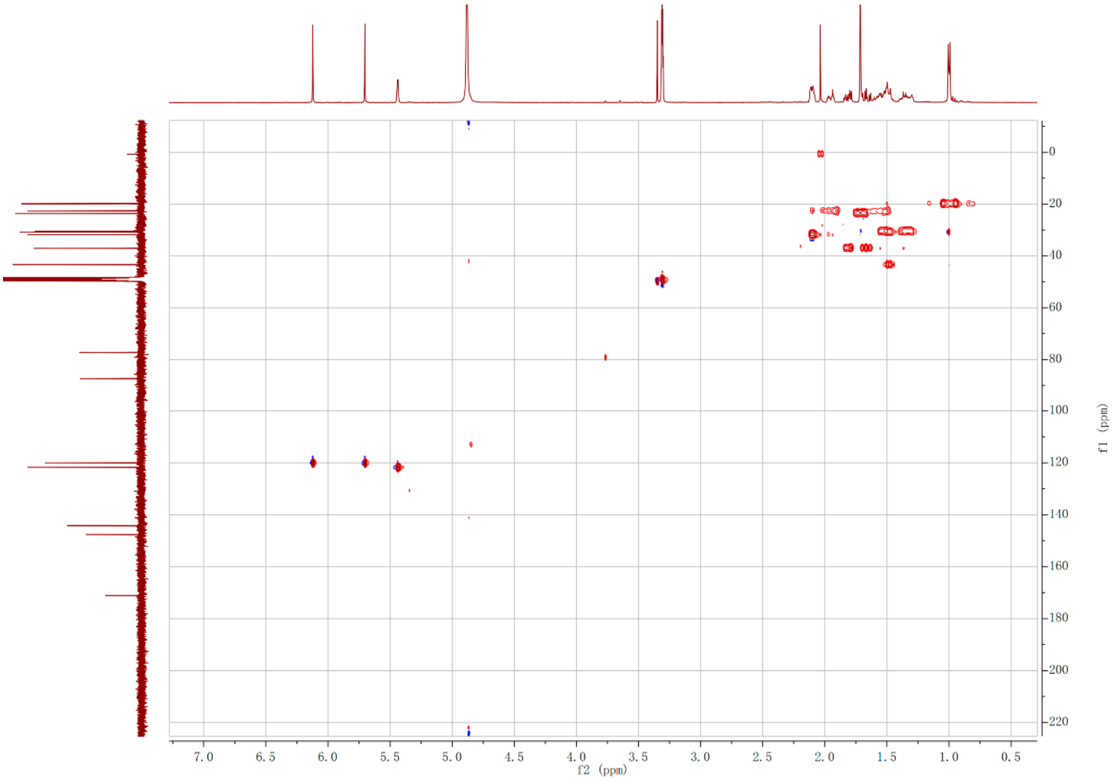

Figure S12. The HMBC spectrum of compound **2** (MeOD, 400 MHz).

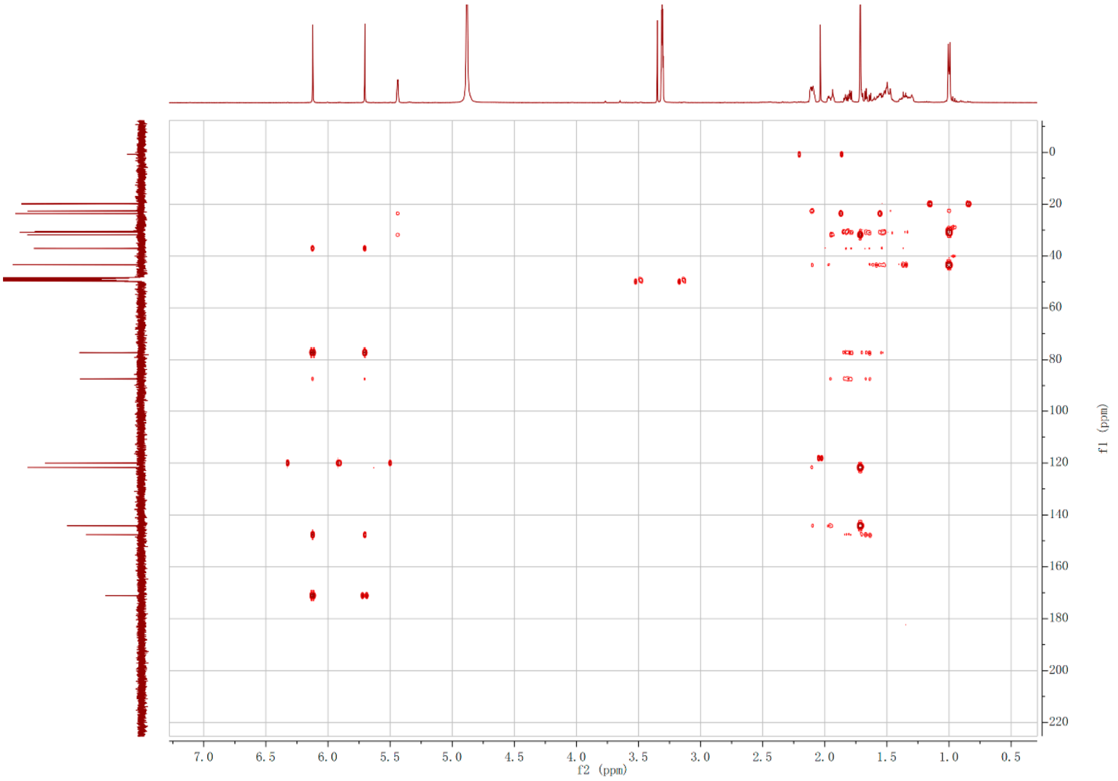

Figure S13. The COSY spectrum of compound **2** (MeOD, 400 MHz).

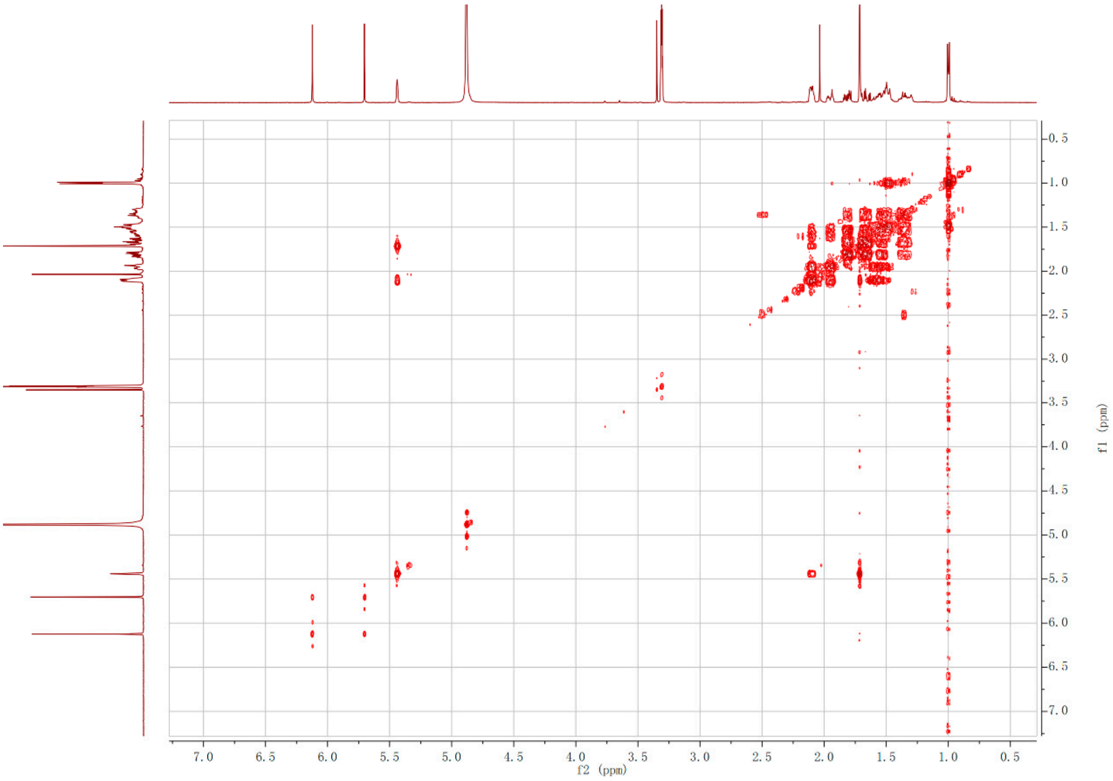

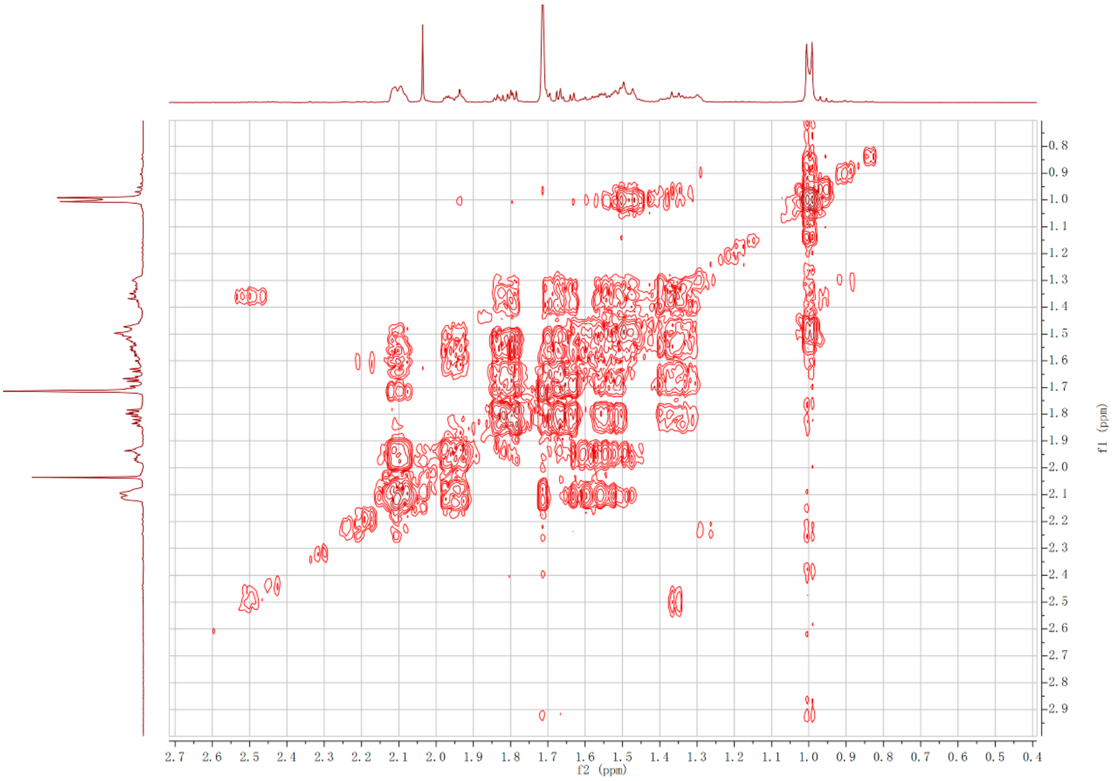

Figure S14. The NOESY spectrum of compound **2** (MeOD, 400 MHz).

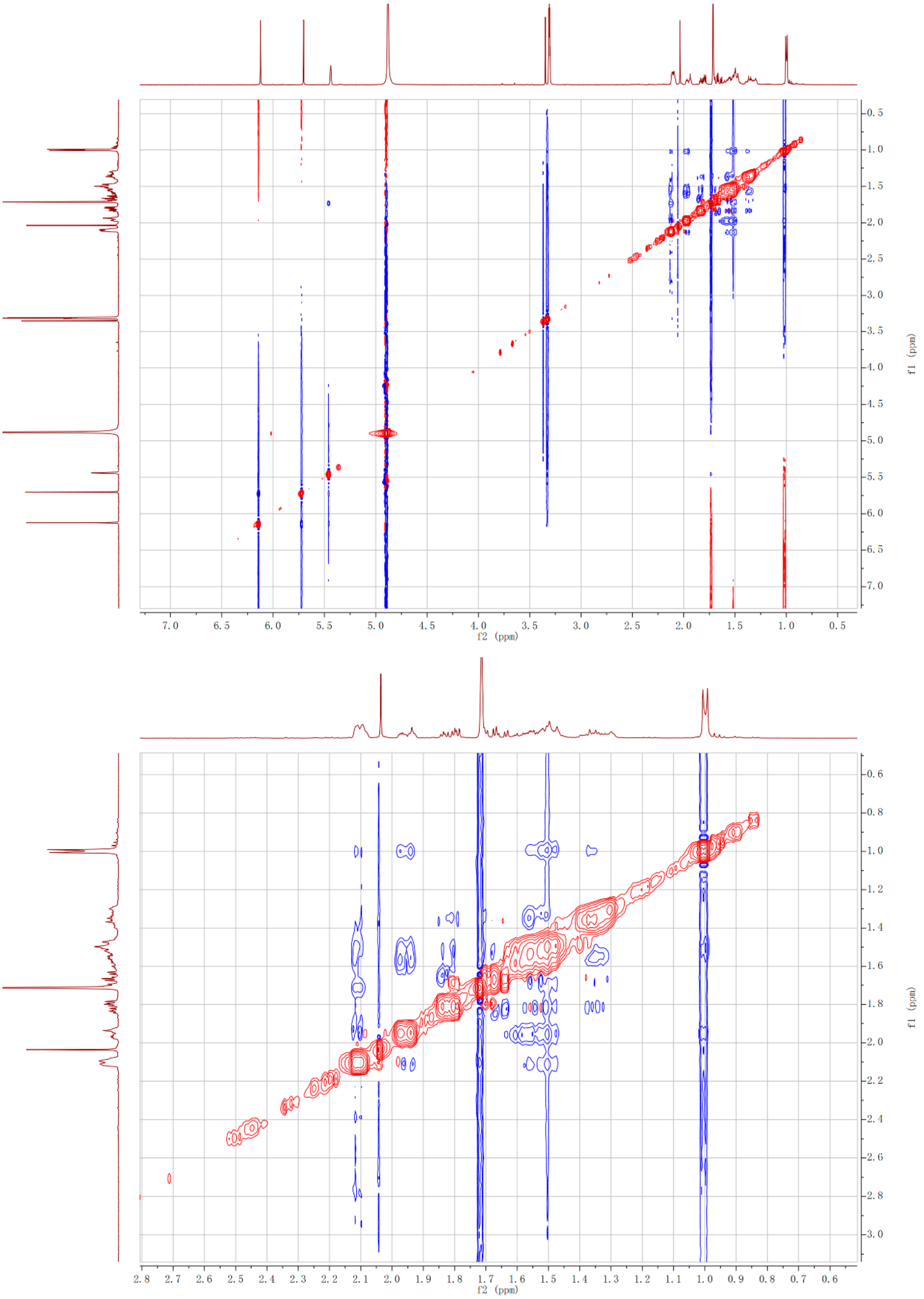

Figure S15. X-ray crystallographic structure of compound **3**

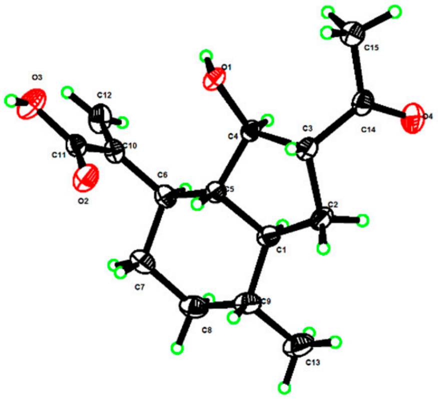

Figure S16. Results for DP4+ analysis of compound **1** with ^{13}C NMR chemical shifts calculated at mPW1PW91/6-31G (d, p) level, (isomer 1 = 6*S**, isomer 2 = 6*R**).

Isomer Nº			1	2	3	4
DP4+ (%)		H data	-	-	-	-
		C data	99.99%	0.01%	-	-
		All data	99.99%	0.01%	-	-
Type	sp2?	Exp	1	2	3	4
C		41.3	40.517775	42.0922181		
C		26.7	37.483075	27.1599786		
C		30.4	32.945875	34.9245684		
C	x	148	148.612775	150.815731		
C		76.9	81.706175	80.5203799		
C		87.9	90.572775	88.6752144		
C		42.4	42.839075	52.2827631		
C		30.1	34.148375	19.1875143		
C		32.1	28.837875	35.5407212		
C		31.1	36.907875	30.842815		
C	x	143.7	142.781275	140.999795		
C	x	172	168.281275	169.404429		
C	x	121.6	126.148675	115.794454		
C		20.4	25.651475	20.4255884		
C	x	113	112.306475	112.823131		

Default parameters	1	2	3	4
sDP4+ (H data)	-	-	-	-
sDP4+ (C data)	98.51%	1.49%	-	-
sDP4+ (all data)	98.51%	1.49%	-	-
uDP4+ (H data)	-	-	-	-
uDP4+ (C data)	99.60%	0.40%	-	-
uDP4+ (all data)	99.60%	0.40%	-	-
DP4+ (H data)	-	-	-	-
DP4+ (C data)	99.99%	0.01%	-	-
DP4+ (all data)	99.99%	0.01%	-	-

编号	实验		No	R_opt	No	S_opt	R校正结果	R校正误差	S校正结果	S校正误差
1	41. 3	1	1	40. 52	1	42. 09	37. 24	-4. 06	41. 22	-0. 08
2	26. 7	2	8	37. 48	8	27. 16	34. 11	7. 41	26. 01	-0. 69
3	30. 4	3	9	32. 95	9	34. 92	29. 43	-0. 97	33. 92	3. 52
4	148. 0	4	10	148. 61	10	150. 82	148. 73	0. 73	151. 99	3. 99
5	76. 9	5	11	81. 71	11	80. 52	79. 73	2. 83	80. 37	3. 47
6	87. 9	6	2	90. 57	2	88. 68	88. 87	0. 97	88. 68	0. 78
7	42. 4	7	3	42. 84	3	52. 28	39. 64	-2. 76	51. 60	9. 20
8	30. 1	8	4	34. 15	4	19. 19	30. 67	0. 57	17. 88	-12. 22
9	32. 1	9	5	28. 84	5	35. 54	25. 20	-6. 90	34. 54	2. 44
10	31. 1	10	6	36. 91	6	30. 84	33. 52	2. 42	29. 76	-1. 34
11	143. 7	11	12	142. 78	12	141. 00	142. 72	-0. 98	141. 99	-1. 71
12	172. 0	12	14	168. 28	14	169. 40	169. 02	-2. 98	170. 92	-1. 08
13	121. 6	13	13	126. 15	13	115. 79	125. 56	3. 96	116. 31	-5. 29
14	20. 4	14	7	25. 65	7	20. 43	21. 91	1. 51	19. 14	-1. 26
15	113. 0	15	15	112. 31	15	112. 82	111. 29	-1. 71	113. 28	0. 28

Figure S17. Correlation coefficients and DP4+ analysis between experimental and calculated ^{13}C NMR chemical shifts of isomer 1 = 6*S** and isomer 2 = 6*R**

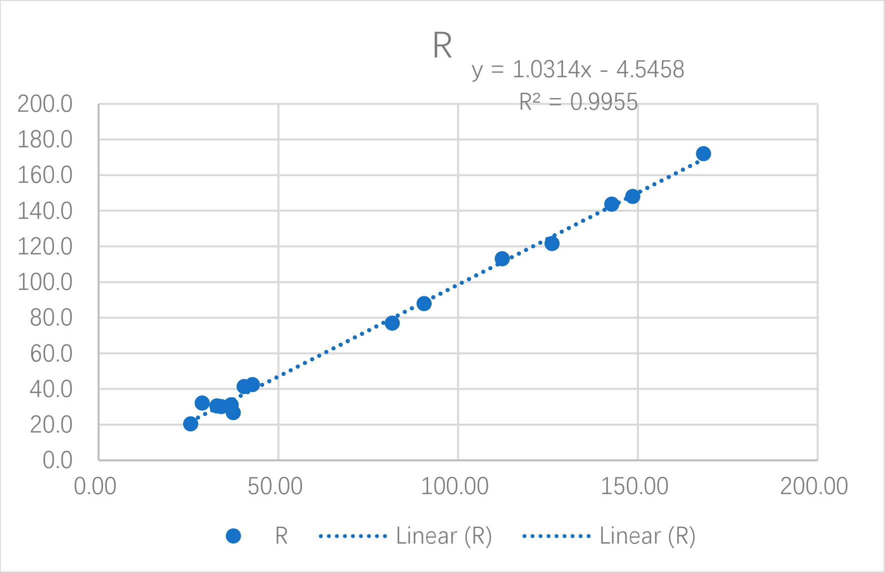

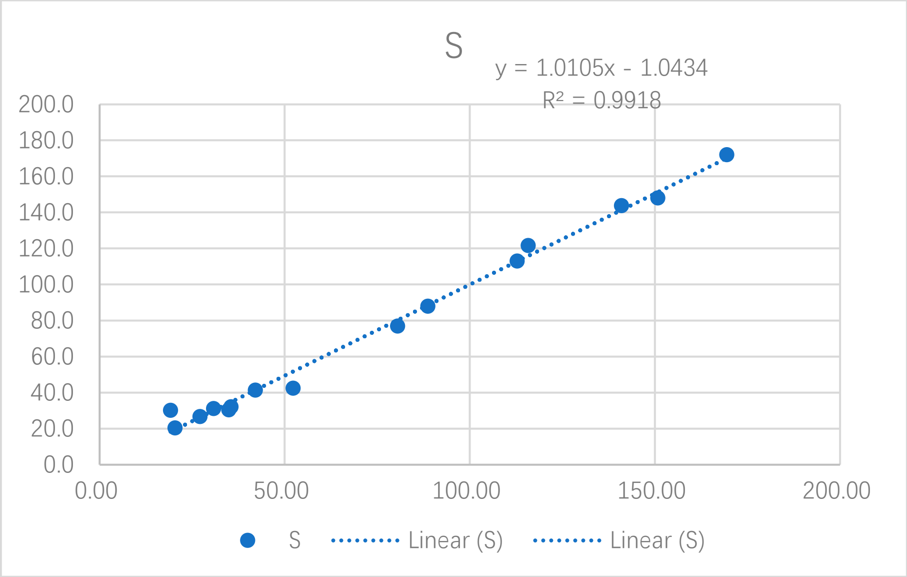

Figure S18. Results for DP4+ analysis of compound **2** with ^{13}C NMR chemical shifts calculated at mPW1PW91/6-31G (d, p) level, (isomer 1 =6*R**, 7*R**, isomer 2 = 6*R**, 7*S**, isomer 3 = 6*S**, 7*R**, isomer 4 = 6*S**, 7*S**).

Isomer Nº			1	2	3	4
DP4+ (%)		H data	-	-	-	-
		C data	100.00%	0.00%	0.00%	0.00%
		All data	100.00%	0.00%	0.00%	0.00%
Type	sp2?	Exp	1	2	3	4
C		43.4	44.930375	50.821875	40.3192067	42.226475
C		22.5	24.811075	22.291575	23.3974092	31.908875
C		31.7	34.618975	34.991675	29.2501822	33.957275
C	x	144.1	151.954475	145.074275	146.018839	151.112775
C	x	121.6	117.076275	128.129575	119.714057	118.780775
C		87.4	86.273875	89.347275	89.5240063	87.884675
C		77.2	79.498775	83.175175	80.3456604	78.862975
C		37	37.870475	25.620175	29.8386493	32.746275
C		30.4	34.087075	34.735075	31.6646385	25.069275
C		30.8	33.137775	32.565675	33.6537984	37.987275
C	x	147.6	147.011675	144.114375	143.139361	147.360875
C	x	171	167.340975	170.557275	168.845066	167.695275
C	x	119.9	120.144375	116.965275	123.306905	120.865775
C		19.8	21.112575	21.216275	21.2257554	21.858475
C		23.5	26.219275	25.486375	26.0791827	25.738375

Default parameters	1	2	3	4
sDP4+ (H data)	-	-	-	-
sDP4+ (C data)	100.00%	0.00%	0.00%	0.00%
sDP4+ (all data)	100.00%	0.00%	0.00%	0.00%
uDP4+ (H data)	-	-	-	-
uDP4+ (C data)	99.99%	0.00%	0.00%	0.00%
uDP4+ (all data)	99.99%	0.00%	0.00%	0.00%
DP4+ (H data)	-	-	-	-
DP4+ (C data)	100.00%	0.00%	0.00%	0.00%
DP4+ (all data)	100.00%	0.00%	0.00%	0.00%

	编号	实验		No	RR_opt	No	RS_opt	No	SR_opt	No	SS_opt
	1	43. 4	1	1	44. 930375	1	50. 821875	1	40. 31920671	1	42. 226475
	2	22. 5	2	8	24. 811075	8	22. 291575	8	23. 39740916	8	31. 908875
	3	31. 7	3	9	34. 618975	9	34. 991675	9	29. 25018218	9	33. 957275
x	4	144. 1	4	10	151. 954475	10	145. 074275	10	146. 0188392	10	151. 112775
x	5	121. 6	5	11	117. 076275	11	128. 129575	11	119. 7140572	11	118. 780775
	6	87. 4	6	2	86. 273875	2	89. 347275	2	89. 52400628	2	87. 884675
	7	77. 2	7	3	79. 498775	3	83. 175175	3	80. 34566036	3	78. 862975
	8	37. 0	8	4	37. 870475	4	25. 620175	4	29. 83864934	4	32. 746275
	9	30. 4	9	5	34. 087075	5	34. 735075	5	31. 66463846	5	25. 069275
	10	30. 8	10	6	33. 137775	6	32. 565675	6	33. 65379836	6	37. 987275
x	11	147. 6	11	12	147. 011675	12	144. 114375	12	143. 1393609	12	147. 360875
x	12	171. 0	12	14	167. 340975	14	170. 557275	14	168. 8450661	14	167. 695275
x	13	119. 9	13	13	120. 144375	13	116. 965275	13	123. 3069048	13	120. 865775
	14	19. 8	14	7	21. 112575	7	21. 216275	7	21. 22575536	7	21. 858475
	15	23. 5	15	15	26. 219275	15	25. 486375	15	26. 07918266	15	25. 738375

Figure S19. Correlation coefficients and DP4+ analysis between experimental and calculated ^{13}C NMR chemical shifts of isomer 1 =6*R**, 7*R**, isomer 2 = 6*R**, 7*S**, isomer 3 = 6*S**, 7*R**, and isomer 4 = 6*S**, 7*S**.

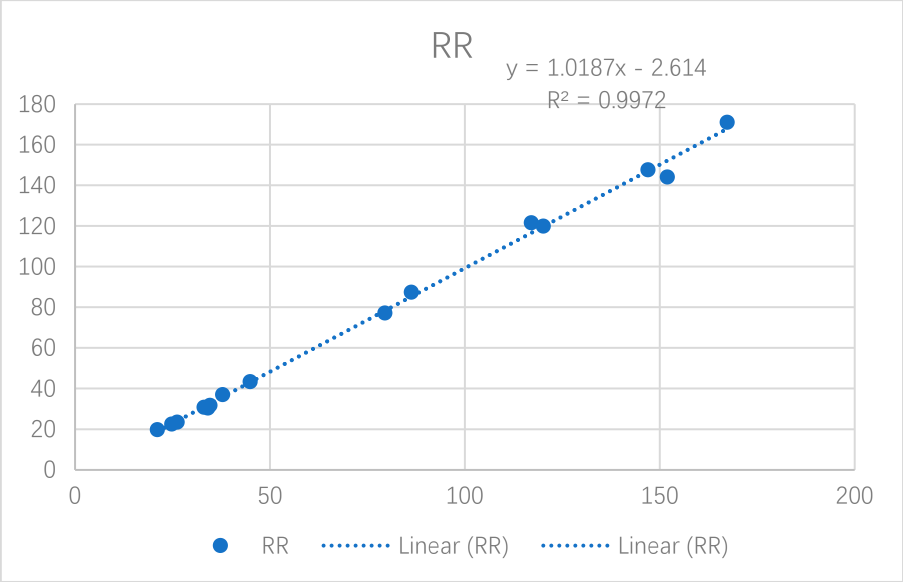

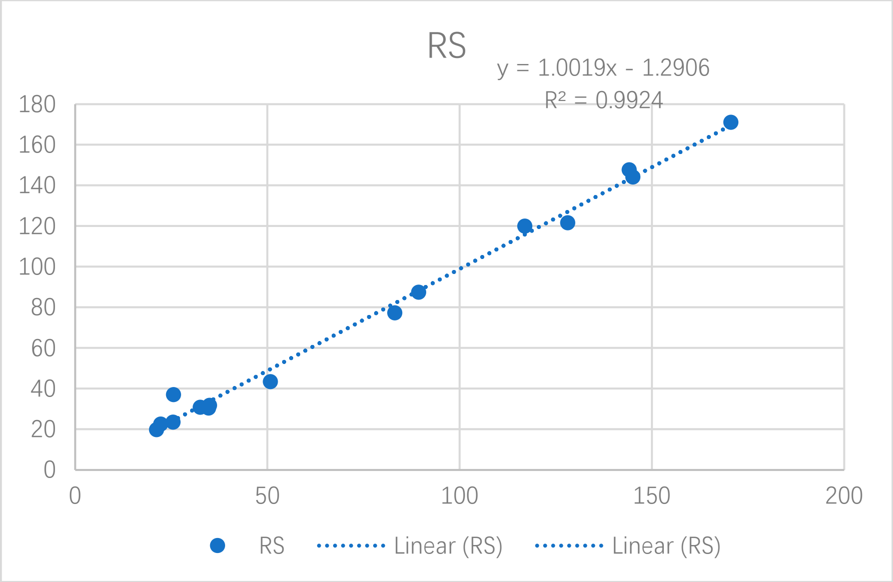

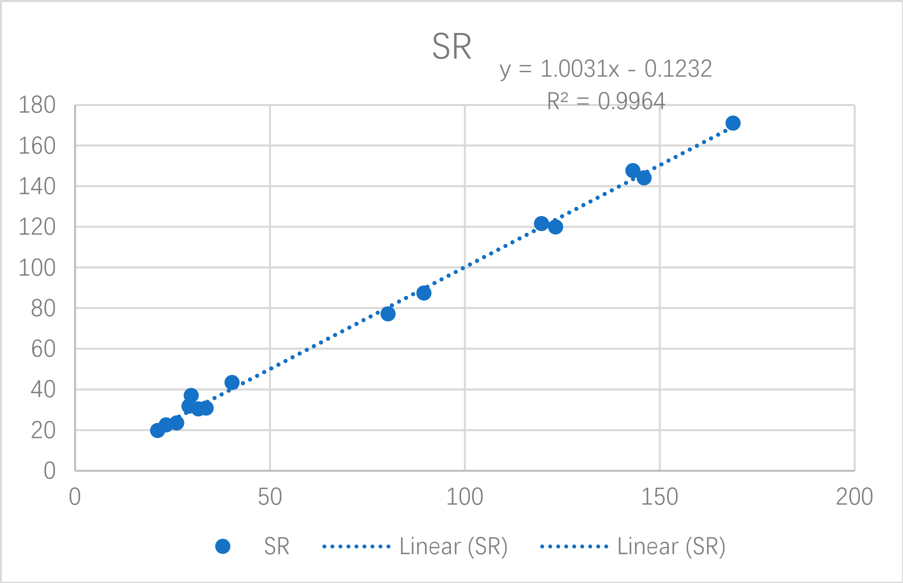
SR
y = 1.0031x - 0.1232
R² = 0.9964
180
160
140
120
100
80
60
40
20
0
0
50
100
150
200
SR
Linear (SR)
Linear (SR)

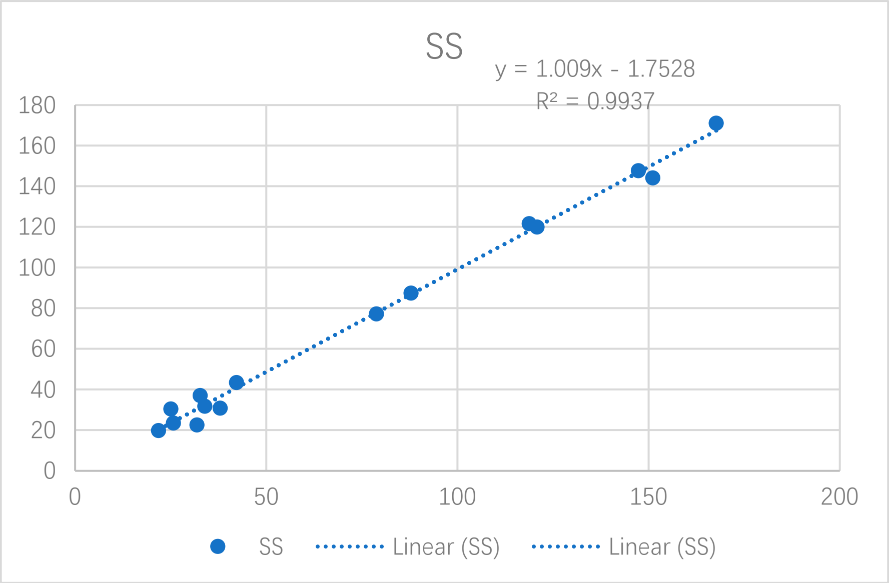
SS
y = 1.009x - 1.7528
R² = 0.9937
180
160
140
120
100
80
60
40
20
0
0
50
100
150
200
SS
Linear (SS)
Linear (SS)
